# Functionalizing Nonfunctional Surfaces: Creation of Metal Oxide Nanopatterns on High-Performance Polymers via Self-Assembly of PS-*b*-PEO

**DOI:** 10.1021/acsami.5c04225

**Published:** 2025-04-09

**Authors:** Jhonattan Frank Baez Vasquez, Aislan Esmeraldo Paiva, Sajan Singh, Sherly Acosta-Beltrán, Alberto Alvarez Fernandez, Michael A. Morris

**Affiliations:** AMBER Research Centre/School of Chemistry, Trinity College Dublin, Dublin D02W085, Ireland

**Keywords:** high-performance polymers, block copolymer self-assembly, metal oxide nanopatterns, surface functionalization, nanostructures, surface modification techniques, nanopatterning methodologies

## Abstract

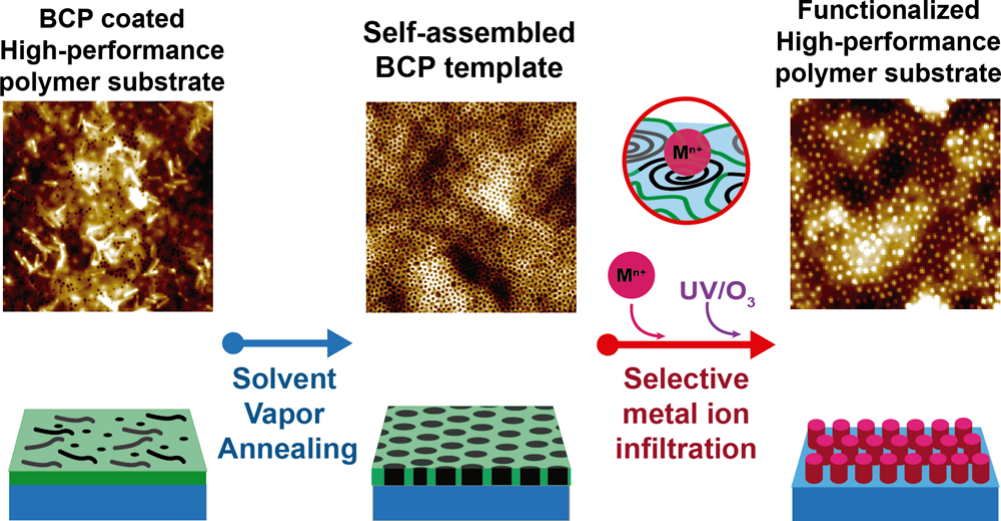

High-performance polymers are pivotal for a wide range of applications due to their excellent mechanical, chemical, and thermal properties. This work introduces, for the first time, a block copolymer (BCP) self-assembly method to modify the surfaces of different high-performance polymers. Using highly ordered poly(styrene-*b*-ethylene oxide) (PS-*b*-PEO) thin films as templates, metallic oxide nanopillars (Al_2_O_3_, Ag_2_O, MgO, CaO, and TiO_2_) with a 20 nm average diameter were fabricated. These were created on high-performance polymer substrates, specifically, polyetheretherketone (PEEK), carbon fiber-reinforced polyetheretherketone (CFPEEK), and ultrahigh molecular weight polyethylene. This method addresses the low chemical activity of these polymeric substrates, offering a cost-effective, scalable solution to produce their surface functionalization. Characterization via atomic force microscopy, scanning electron microscopy, and X-ray photoelectron spectroscopy validate the structure and composition of the nanostructured surfaces. The significance of BCP self-assembly is emphasized as an effective and versatile approach for the nanoscale tailoring of surface properties in high-performance polymers. This process offers a straightforward method with low technological and energetic costs, paving the way for the extensive surface modification of large areas. The implications of this work extend to various sectors, including biomedical devices, sensors, and electronics, showcasing the broad applicability of this nanoscale tailoring technique.

## 1 Introduction

High-performance polymers exhibit excellent mechanical, thermal, and chemical resistance properties.^1,2^ This, combined with their versatility, has led to their widespread adoption in various industries, such as automotive, aerospace, electronics, and medical applications.^3−5^ Interestingly, the performance of these polymers can be further enhanced through surface modification strategies, which aim to improve properties such as wear resistance,^6,7^ biocompatibility,^8,9^ or adhesion to other materials,^10,11^ while their bulk properties remain unaltered. In this context, techniques such as ultraviolet light (UV) and plasma treatments,^12^ wet chemical modification or electrospraying,^13^ have shown their applicability to tailor the surface properties of these polymers to meet specific application requirements. Among the available surface modification strategies, coating processes are a very effective solution for improving material properties. Usually, these methods generate coatings with layers in the order of nanometers to micrometers, covering large surface areas with small amounts of coating material.^14^

Coating methods offer a wide variety of deposition molecules and improved properties on polymer substrates.^12,14^ For example, the introduction of molecules like chitosan^15^ or polyurethanes,^16,17^ enables the production of long-term wettability modifications, while the use of metal alkoxides^18^ or poly(acrylic acid) (PAA)^19^ allows for increased abrasion resistance properties. Also, the use of poly(ethylene glycol) (PEG) and PEG derivatives has been commonly reported for the production of antibacterial polymer surfaces.^16,20^ The inclusion of poly(3,4-ethylenedioxythiophene) (PEDOT)^21^ or metallic particles^22^ on polymer surfaces can increase its electrical conductivity. Additionally, the coating with nanodiamond fibers^23^ can improve the surface hardness. Among the variety of options for coatings, metal oxides play an important role. Metal oxides of titanium, aluminum, zinc, zirconium, or silicon have been reported for providing UV light resistance,^24^ abrasion resistance,^25^ or improved biointegration.^26−28^

In addition to the chemical modification of the surface, the nanostructure of the coating (including roughness, shape, and thickness) is equally important.^12^ While coating methods such as sol–gel deposition, spraying (e.g., cold/heat spraying, electrospraying), wet chemical reactions, or plasma treatment can produce chemical modifications, they often fail to generate surfaces with well-ordered nanostructures and the benefits of material patterning on the nanoscale.^14^ However, nanostructuring has proven to be crucial for the successful introduction of several nanopatterned materials in a wide range of applications.^12^ Nanostructured coatings have been instrumental in advanced fields such as optoelectronics, where precise control over the coating’s nanostructure allows for enhanced light absorption,^29,30^ improved energy conversion efficiency,^31,32^ and optimized photon management.^33^ Moreover, nanostructured coatings have demonstrated significant improvements in data storage capabilities, enabling higher data density,^34^ and improved read/write performance.^35^ In addition, the antimicrobial activity and biointegration of high-performance polymers can be greatly enhanced through nanostructuring.^36−38^ The controlled nanostructure provides a larger surface area,^37^ allowing increased contact with microorganisms and cells and improving the efficiency of antimicrobial agents. This has promising applications in healthcare-related environments, where preventing the spread of infections is crucial. Furthermore, nanostructured coatings have been extensively employed in the development of biosensors, facilitating highly sensitive and selective detection of biological molecules.^39,40^ The nanostructured surfaces enable efficient immobilization of biomolecules, improved signal transduction, and enhanced interactions with target analytes, leading to enhanced sensing performance.^41,42^ However, the majority of these studies have focused on metallic or inorganic surfaces. Therefore, the development of cost-effective and accessible methods for producing nanostructured coatings for polymers is of critical significance for further advances in these fields.

In addition to these more conventional methods, a surface nanostructuring technique has recently been developed and applied to silicon and glass substrates for potential applications in areas such as electronics,^43^ optical devices,^44^ functional materials,^45^ catalysis,^46^ and more. This technique involves the use of block copolymers (BCPs), polymers consisting of two or more chemically distinct homopolymer blocks (i.e., polymer chains) that are covalently bonded together.^47^ BCPs can self-assemble to form well-ordered periodic microdomains at molecular length scales (e.g., spheres on a cubic lattice, hexagonally close-packed cylinders (HCP), and alternating lamellae), producing self-organized films.^48−50^ Thus, with the appropriate selection of the BCP composition and the Flory–Huggins interaction parameter (χ) between blocks,^48,51^ BCP films can be used as templates to create nanopatterned surfaces through the inclusion of metal ions in the selected domain of the BCP.^52−56^ This ability makes BCP patterning a faster, simpler, and cost-effective way to generate metal oxide surface features of nanometer dimensions with high surface area and order, compared to other coating techniques.^57^

In this work, we propose the creation of metal oxide nanopillar-like coatings by using BCPs for surface nanostructuring on high-performance polymers, specifically polyetheretherketone (PEEK), carbon fiber reinforced PEEK (CFPEEK), and ultrahigh molecular weight polyethylene (UHMWPE). These three materials are well-known for their high mechanical strength, biocompatibility, and wear resistance, properties which allows them to be widely used in joint replacements, spinal implants, and other orthopedic applications.^58−61^ We demonstrate a simple process of dip-coating followed by a solvent vapor annealing step to form a self-organized film of poly(styrene-*b*-ethylene oxide) (PS-*b*-PEO) on these polymer substrates. We also show how these PS-*b*-PEO films can be used as templates to generate coatings of nanopillar-like features of different metal oxides on these polymeric materials. To the best of our knowledge, this is the first successful work showing the practicality of these methods on high-performance polymers, and we believe this work opens new possibilities for the application of BCP templating technology on other types of polymeric substrates for various fields, such as biomedical devices, sensors, and electronics.

## 2 Results and Discussion

**Figure 1:**
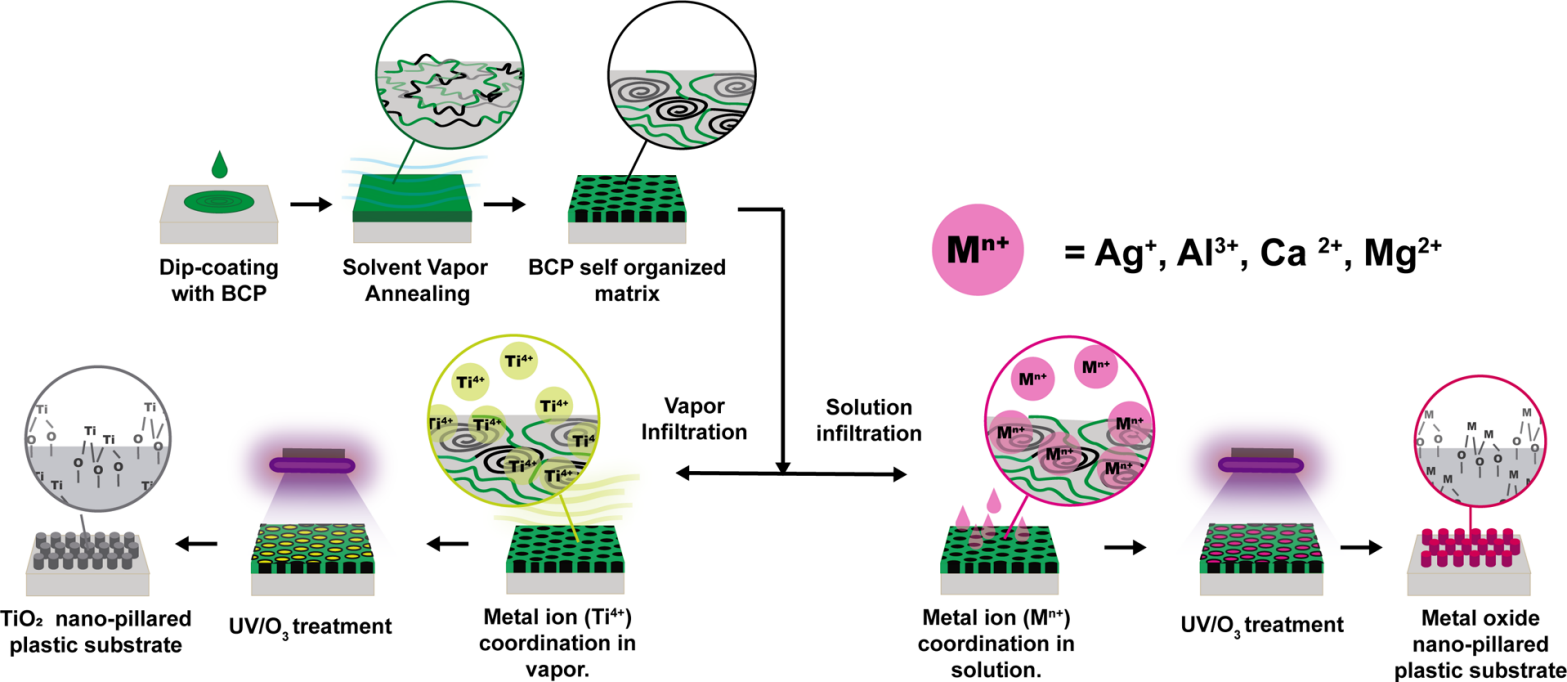
Process diagram for the deposition of metal oxides onto polymeric substrates.

The high-performance polymer surfaces were nanopatterned following the methodology illustrated in Figure 1. As a first step, the polymer substrate is covered with a PS-*b*-PEO film by dip-coating. The substrate is then subjected to a solvent vapor annealing (SVA) step using toluene. Toluene, a preferentially selective solvent for the PS block of the PS-*b*-PEO BCP used in this study (see solubility data in Table S1 from SI), was employed during the solvent annealing step to promote microphase separation. This resulted in the formation of a self-organized thin film with PEO domains forming vertical or parallel-aligned cylinders within a PS matrix.^51^ It is worth noting that microphase separation can also be induced using nonselective solvents, depending on the polymer system and processing conditions.

The subsequent step is the infiltration of metallic ions into the PEO domains of the PS-*b*-PEO film (i.e., the coordination of the ions with the oxygen present in the PEO domains^55^). In the case of Ti^4+^ ions, this was conducted via chemical vapor deposition (CVD) using TTIP, while for the other metallic ions tested (Al^3+^, Ag^+^, Ca^2+^, and Mg^2+^), the infiltration was performed by the immersion of the substrate in an ethanolic solution of each metallic ion. Finally, the infiltrated substrates were passed through a UVO treatment, which produced the degradation and removal of the BCP film, leaving a nanostructured surface.

### 2.1 Self-Assembled PS-*b*-PEO Thin Films

**Figure 2:**
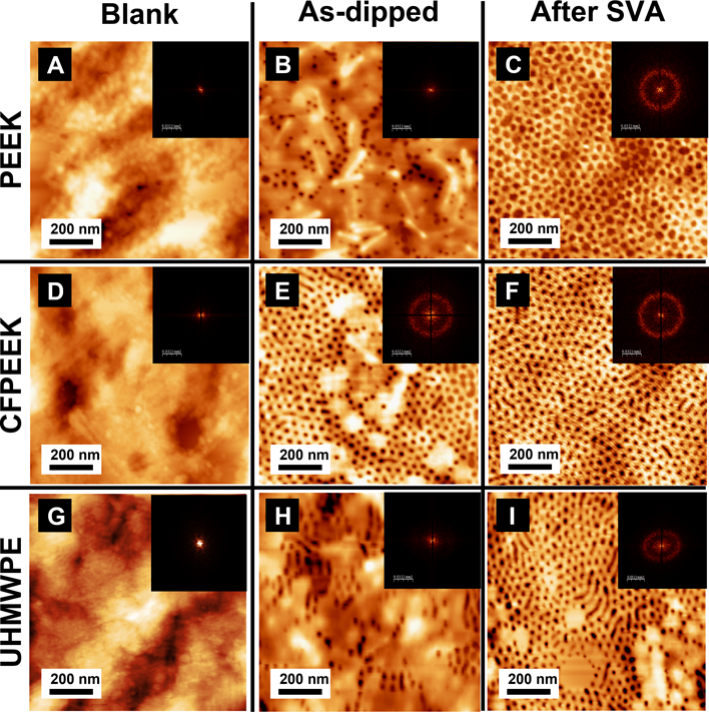
Effect of the SVA. Letters from (A) to (I) corresponds to AFM images, Insets correspond to FFT images for each AFM image.

Figure 2 displays AFM images of PEEK, CFPEEK, and UHMWPE substrates during the dip-coating to solvent vapor annealing (SVA) steps (see Figure 1). All coated substrates exhibited evidence of microphase separation following the dip-coating of PS-*b*-PEO, resulting in the formation of PEO HCP vertical cylinders within a PS matrix.

**Figure 3:**
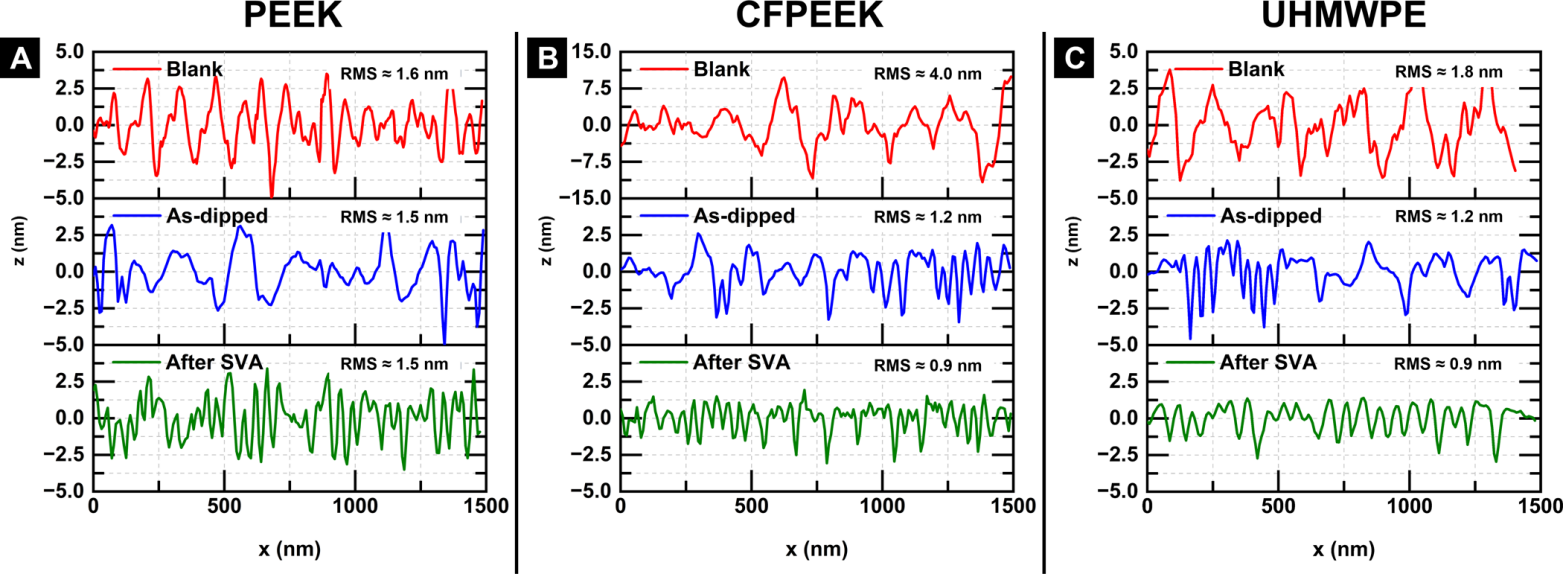
Roughness profiles for PEEK (A), CFPEEK (B), and UHMWPE (C) in the different stages of the production of self-assembled PS-*b*-PEO thin film coatings. (The roughness profiles are determined from the images shown in Figure 2, the zones of each AFM image used can be found in Figure S2 in SI.).

On PEEK substrates, PEO vertical cylinders are observable as black dots (Figure 2B), showing a random distribution within the film (see inset in Figure 2B). In contrast, CFPEEK demonstrates a more regular microphase separation process, featuring short-range ordered PEO vertical cylinders covering a significant portion of the imaged area (Figure 2E). Similarly, UHMWPE exhibits both vertical and parallel PEO cylinders, with parallel cylinders being dominant configuration and lacking symmetrical distribution (Figure 2H). These results suggest that the substrate influences the free energy of the PS-*b*-PEO film. Although this influence is minor on PEEK and UHMWPE substrates, CFPEEK, with the presence of carbon fibers, seems to induce a reduction in surface free energy.^48^ This reduction leads to a higher degree of microphase separation in the as-dipped substrate compared to the as-dipped PEEK. Furthermore, the roughness profile of the AFM micrographs reveals that PEO vertical cylinders produced by microphase separation in all as-dipped substrates have a depth of approximately 2.5 nm. The corresponding RMS values are approximately 1.5, 1.2, and 1.22 nm for PEEK, CFPEEK, and UHMWPE coated substrates, respectively (see Figure 3).

After the PS-*b*-PEO dip-coating step, all samples underwent solvent annealing with toluene at 50 °C for 1 h (12.3 kPa saturation pressure, derived from Antoine’s equation^62^) as described in the experimental section. The AFM results in Figure 2 depict the complete microphase separation in the BCP film across all substrates, resulting in a notable increase in the number of vertically aligned PEO cylinders compared to the as-dipped films (see Figure 2C,F,I).

**Figure 4:**
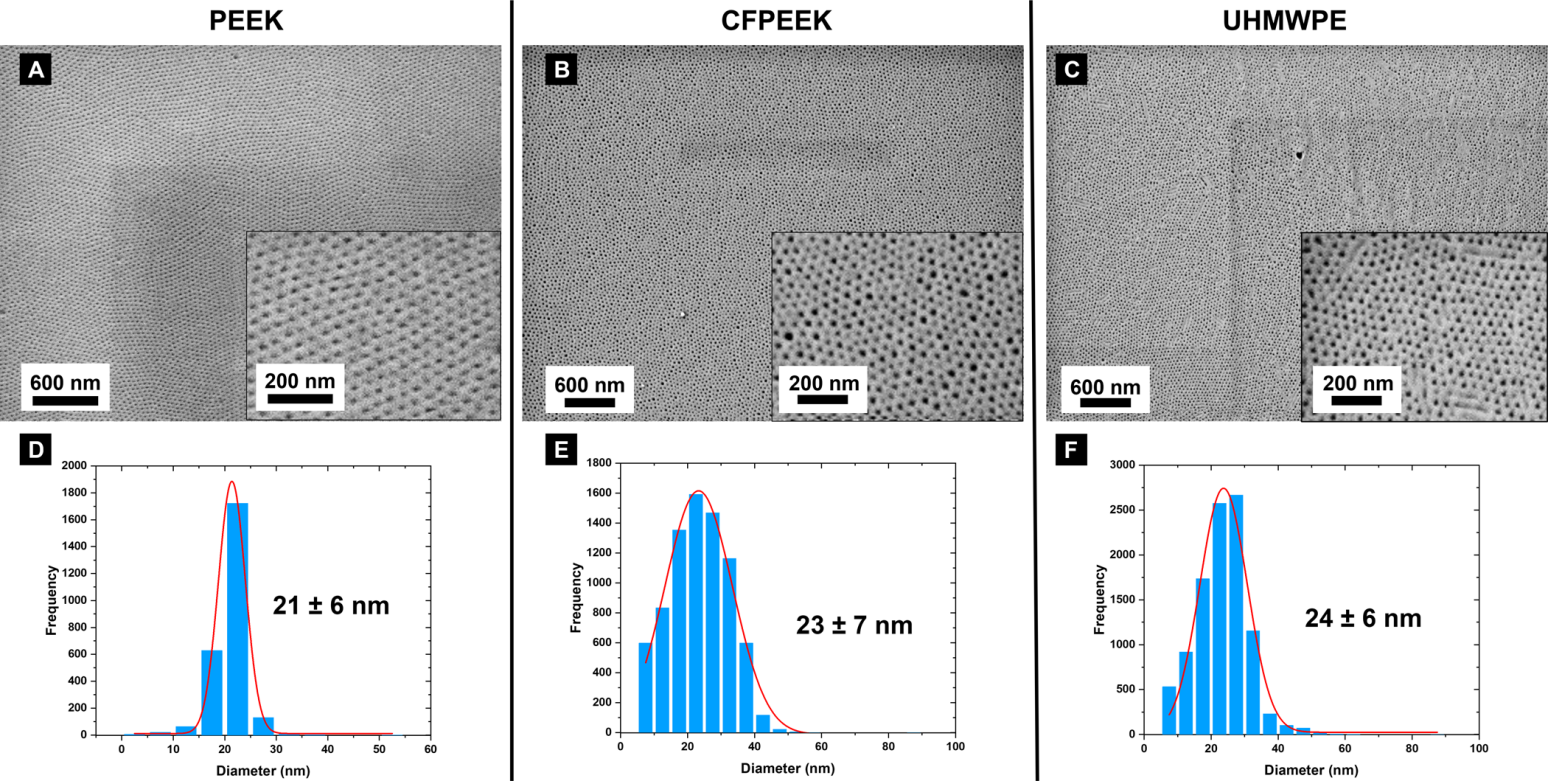
SEM images of PEO vertical cylinders on PS-*b*-PEO coated substrates after SVA: (A) PEEK, (B) CFPEEK, and (C) UHMWPE. Each panel displays the SEM image (top) and a zoomed-in region (inset). Panels (D–F) show the corresponding size distribution histograms calculated from the SEM images for (A–C), respectively, including the extracted average diameters.

To validate these observations on a larger scale, SEM analysis was performed, confirming the consistent formation of long-range HCP vertical cylinders in the PS-*b*-PEO film (see Figure 4 (top)). Size distribution histograms derived from the SEM images (Figure 4 (bottom)) illustrate that the BCP films on the three tested substrates exhibit PEO vertical cylinders with diameters predominantly around ≈20 nm. Notably, PEO domains on PEEK substrates demonstrate higher uniformity around this value compared to those on CFPEEK and UHMWPE, where a broader range of diameters is observed. This can be attributed to the specific interactions between the PEO blocks and PEEK surface that promote self-organization of the polymer into more ordered structures. The solvent vapor likely facilitates the reorganization of the polymer chains, helping to align the PEO cylinders on the PEEK substrate.

**Figure 5:**
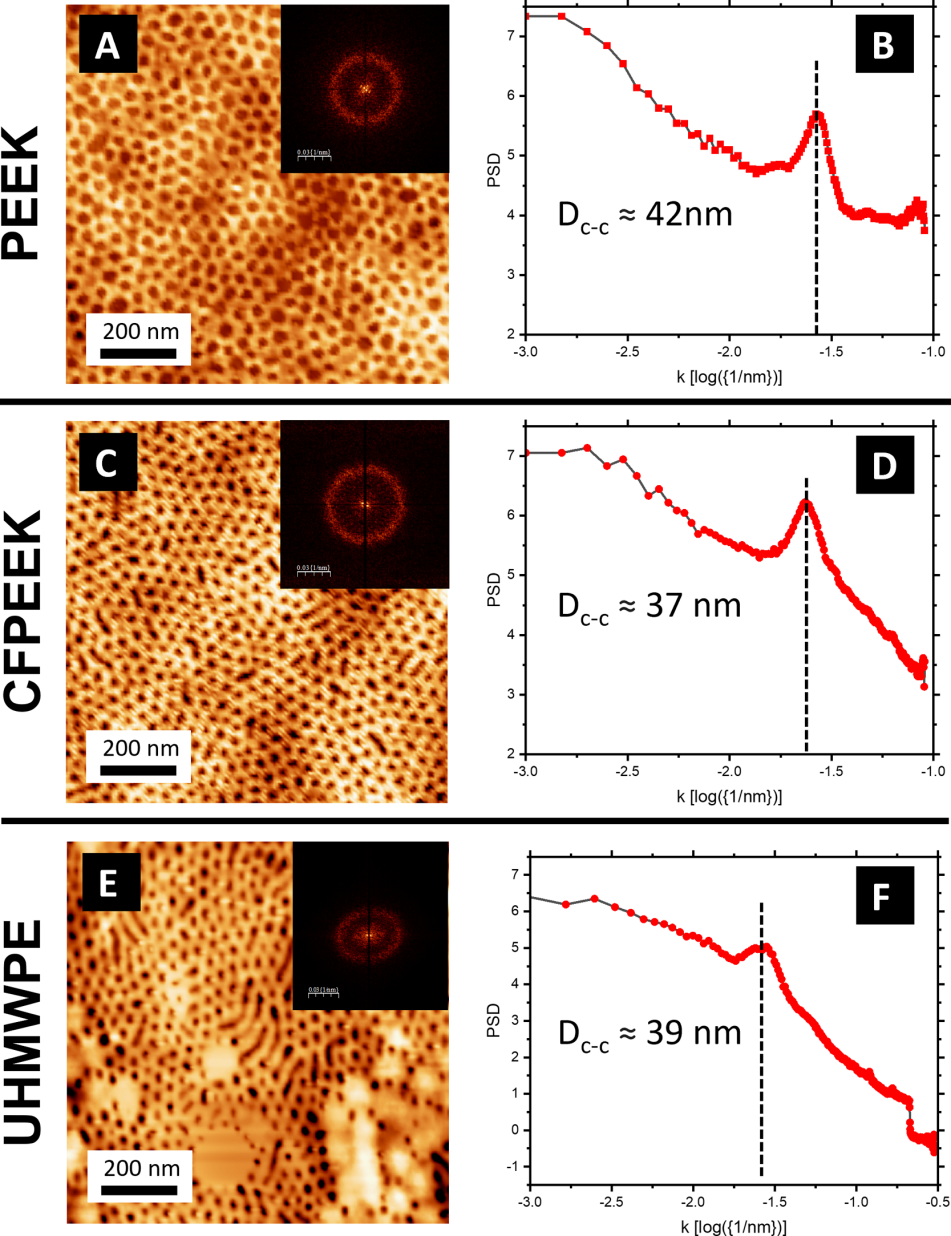
AFM images (left) and corresponding Power Spectral Density (PSD) functions (right) for PS-*b*-PEO coated substrates after the SVA step: (A, B) PEEK, (C, D) CFPEEK, and (E, F) UHMWPE. PSD plots are calculated from the Fast Fourier Transform (FFT) images shown as insets in the AFM panels and are used to extrapolate the center-to-center distance (*D*_*C*–*C*_) between the nanopores. AFM images with particle masks used for *D*_*C*–*C*_ calculation are provided in Figure S1 in the Supporting Information.

Furthermore, the center-to-center distance (*D*_*C*–*C*_) and the RMS roughness values vary among the substrates (see Figures 3 and 5). For instance, PEO vertical cylinders in PS-*b*-PEO films on CFPEEK and UHMWPE exhibit *D*_*C*–*C*_ values of approximately 37 nm, whereas on PEEK this distance increases to about 42 nm. The RMS values obtained for the microphase-separated films are approximately 1.5, 0.9, and 0.9 nm on PEEK, CFPEEK, and UHMWPE, respectively. In addition, the PEO vertical cylinders on PEEK are substantially deeper (≈3 nm) than those on CFPEEK or UHMWPE (≈1.5 nm).

Although BCP self-assembly typically dictates the intrinsic domain spacing, substrate-related factors can still introduce variations in *D*_*C*–*C*_. In particular, differences in substrate surface energy may influence the microphase separation process. Based on water and diiodomethane contact angles (Table S3 in the Supporting Information), the calculated surface energies are 21.60 ± 1.29 mJ m^–2^ (PEEK), 32.21 ± 3.28 mJ m^–2^ (CFPEEK), and 32.37 ± 3.04 mJ m^–2^ (UHMWPE).

Because PEEK has the lowest surface energy, it likely interacts more weakly with the PS block in PS-*b*-PEO. By contrast, CFPEEK and UHMWPE—both having higher surface energies—could exhibit stronger interfacial interactions with the PS domains. These enhanced interactions may locally confine the polymer chains and reduce the effective domain spacing, thereby resulting in a smaller center-to-center distance (i.e., ∼37 nm on CFPEEK and UHMWPE vs ∼42 nm on PEEK).

### 2.2 Production of Metal Oxide Nanopillar Films

Self-assembled PS-*b*-PEO films were employed as templates for the fabrication of metal oxide nanopillar-like structures on top of the three polymeric substrates (Figure 1). This is a well-reported nanostructuring procedure that is commonly employed on substrates like Si wafers and In_2_O_3_·(SnO_2_) glasses, with spin-coating being the most frequently used method for the metallic ion infiltration of the PEO domains in the PS-*b*-PEO film.^26,52,53,56^

In this study, we opted to use a simpler method: immersing the PS-*b*-PEO–coated substrates in ethanolic solutions containing the Al^3+^, Ag^+^, Ca^2+^, or Mg^2+^ ions to be infiltrated (Section 3). Nevertheless, for Ti^4+^ ions, we employed a variation of the chemical vapor deposition procedure reported by Giraud et al.,^55^ with TTIP as the metallic ion source. The metal oxide nanopillars were produced by exposing the infiltrated samples to UVO treatment (Section 3). This treatment removes the BCP film, leaving nanopillars formed by the oxidation of the coordinated ions in the PEO domains of the self-assembled PS-*b*-PEO film template (Figure 1), and producing a surface nanostructured material.

**Figure 6:**
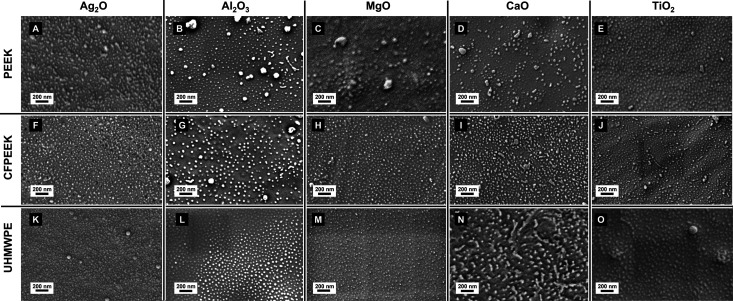
SEM images of metal oxide nanopillar coatings fabricated on PS-*b*-PEO templated (A–E) PEEK, (F–J) CFPEEK, and (K–O) UHMWPE substrates. Columns correspond to different metal oxides: TiO_2_, Al_2_O_3_, MgO, CaO, and Ag_2_O (from left to right). Each image shows the resulting surface morphology following metal ion infiltration and UVO treatment, highlighting the variation in pattern formation across both substrate type and metal oxide composition.

SEM and AFM images confirm the successful production of the desired nanopillar structures in all treated substrates (Figures 6 and S3(SI), respectively), indicating a considerable degree of metal ion infiltration in the PEO vertical cylinders of the microseparated PS-*b*-PEO films. The observed nanopillars, regardless of the substrate or the infiltrated metallic ion, exhibited heights mainly around 20 nm in the AFM analysis (Figure S3 in SI). While we confirm the presence of metal oxides using XPS, SEM, and AFM, we have not quantified the degree of metal ion infiltration. A more detailed quantification of infiltration efficiency could be considered in future studies to further optimize the process.

**Figure 7:**
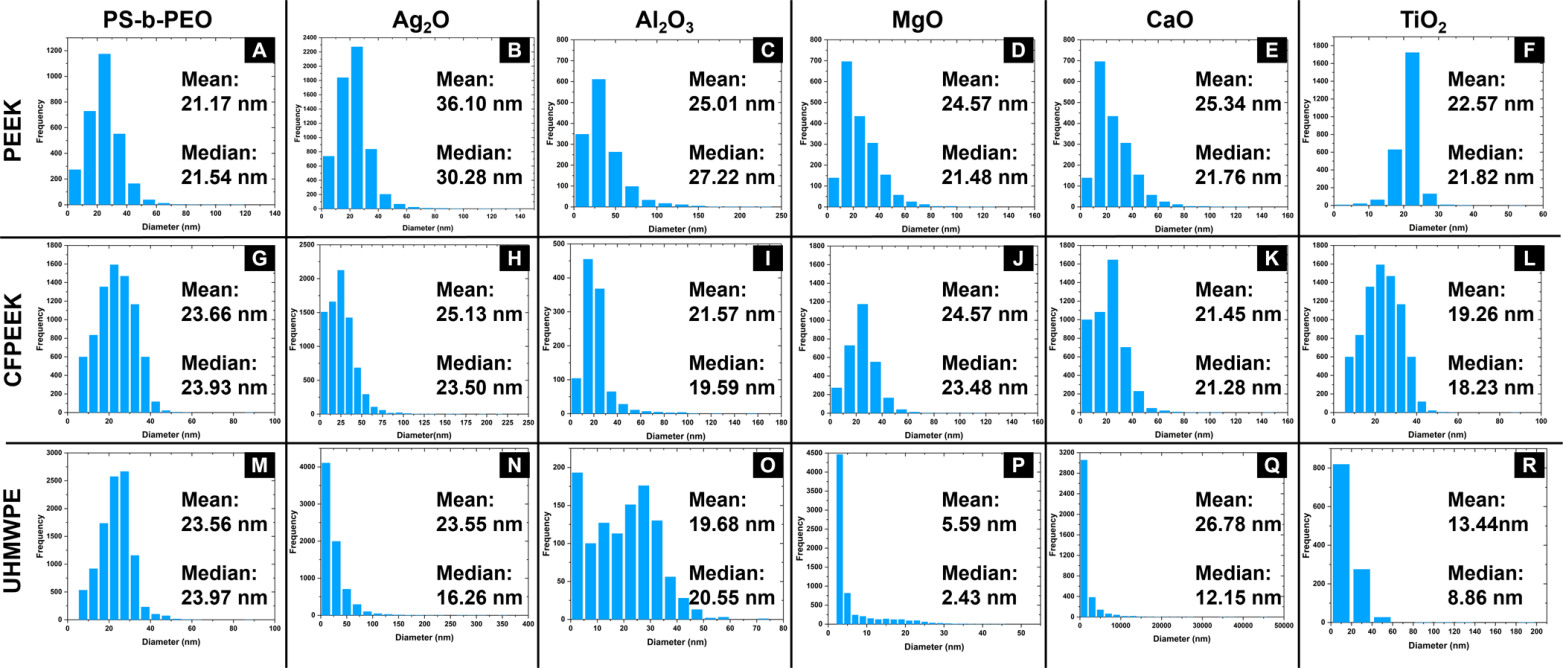
Size distribution histograms for PEEK (A–F), CFPEEK (G–L), and UHMWPE (M–R) coated substrates. Columns represent different coatings: PS-*b*-PEO (A,G,M), Ag_2_O (B,H,N), Al_2_O_3_ (C,I,O), MgO (D,J,P), CaO (E,K,Q), and TiO_2_ (F,L,R). Histograms in panels A, G, and M correspond to the PS-*b*-PEO template before metal infiltration (presented in Figure 4), while the remaining histograms are derived from the SEM images shown in Figure 6. This figure enables an assessment of the pattern transfer fidelity from the BCP template to the final metal oxide nanostructures across different substrates and metal ions.

The formation of nanopillars occurs due to the templating by selective infiltration of metal ions into the microseparated PS-*b*-PEO film and the subsequent removal of BCP and metal ion oxidation. The degree of this transfer can be evaluated by comparing the size distribution of the coated substrates. Our process exhibits a variable level of replication of the original block copolymer pattern, as depicted in Figure 7. For most of the metal ions studied, the mean and median diameters of the metal oxide nanopillars closely resemble the mean and median values of the PEO domain diameter in the microseparated PS-*b*-PEO films.

However, as shown in Figures 6 and 7, particles with diameters in the range of hundreds of nanometers are present to varying degrees in all the evaluated samples. These variations can be attributed to differences in metal ion properties, such as size, solubility, ion’s charge and coordination behavior, and concentrations used in the metal ion source solutions, which differently affect each tested ion, generating a different infiltration degree of the PS-*b*-PEO film template and, consequently, impacting the templating. For instance, Ti^4+^ (TiO_2_) nanopillars appear well-defined, likely due to controlled vapor-phase infiltration, which ensures precise diffusion and templating. In contrast, Ag^+^ (Ag_2_O) and Al^3+^ (Al_2_O_3_) exhibit moderate accuracy but some aggregation, suggesting that solution-phase infiltration may lead to partial overloading.

Extreme cases of this effect are observed in UHMWPE treated with Ca^2+^ ions, exhibiting a high occurrence of larger particles (indicating poor pattern transfer), and PEEK treated with Mg^2+^ ions, which show a low presence of nanopillars and the presence of larger MgO particles, plausibly due to differences in solubility and coordination behavior, which result in uncontrolled deposition and larger aggregates in certain regions.

Moreover, the fact that all the metals evaluated have formed nanopillars, but presented different degrees of larger particle formation across the same ion evaluated at distinct substrates, suggests an influence of the substrate’s surface in the process of infiltration or the degradation of the BCP template by the UVO treatment, probably by influencing the metal ions’ affinity to the PEO domains. Understanding the influence of these larger aggregates on the long-term stability and durability of nanopatterned surfaces is essential, particularly for applications like biomedical implants or sensors, where stable and homogeneous functionalization is critical for properties such as biointegration, antibacterial activity, and sensor performance—including resolution, signal-to-noise ratio, and signal stability. Additionally, particle aggregation could impact the scalability of this method by affecting the repeatability and consistency of nanopatterned surface properties. Consequently, future studies should aim at optimizing infiltration conditions to mitigate particle aggregation and systematically assess the long-term morphological and chemical stability of these nanopatterns under realistic environmental and operational conditions.

**Figure 8:**
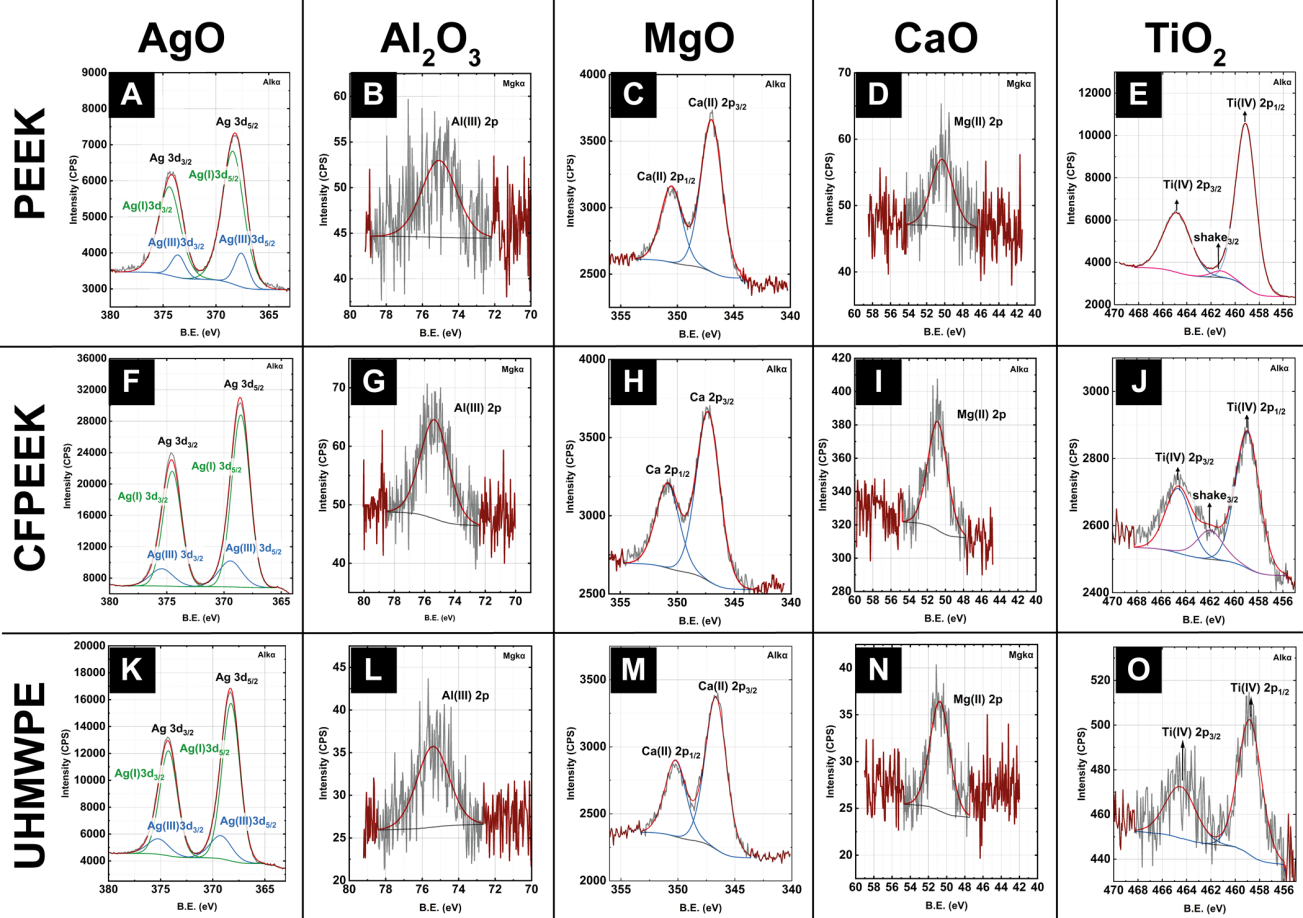
XPS fitted spectra for metal oxide nanopillar coatings on PS-*b*-PEO templated substrates: PEEK (A–E), CFPEEK (F–J), and UHMWPE (K–O). Columns correspond to different metal oxides: Ag_2_O, Al_2_O_3_, MgO, CaO, and TiO_2_ (left to right). Each spectrum confirms the chemical composition of the deposited nanopillars. Peaks were fitted based on reference binding energies to identify characteristic oxidation states.

To confirm the composition of the deposited nanopillar coatings on PEEK, CFPEEK, and UHMWPE, XPS analysis was carried out. Figure 8 shows the XPS core scans obtained, the obtained components and their respective assignments determined in the fitting process for all the XPS spectra are presented in Table S2 in the SI. All the XPS core scans demonstrate the successful formation of Ag, Al, Ca, Mg, and Ti oxides on the polymer substrates after the UVO treatment. Samples infiltrated with the AgNO_3_ ethanolic solution show peaks around ≈368.2 and ≈369 eV (Figure 8A,F,K), corresponding to a 3*d*_5/2_ transition present in Ag oxides, specifically to oxides containing Ag(I) and Ag(III).^63,64^ On the other hand, samples infiltrated using Al^3+^, Ca^2+^, and Mg^2+^ nitrates show peaks at ≈75, ≈347, and ≈50.5 eV, respectively, corresponding to Al(III)(2p) (Figure 8B,G,L), Ca(II)(2*p*_3/2_) (Figure 8D,I,N) and Mg(II)(2p) (Figure 8C,H,M) electronic transitions.^64−67^ Finally, samples treated with TTIP as a source of Titanium ions, present peaks around ≈459 eV (Figure 8E,J,O), corresponding to electrons in the 2*p*_3/2_ level of Ti(IV) present in TiO_2_.^55,64^

The methodology presented here demonstrates the successful nanostructuring of high-performance polymers through the deposition of metal oxide nanopillars. It also elucidates the behavior of dip-coated PS-*b*-PEO films on these substrates, confirming their suitability as effective patterning templates.

Moreover, this approach offers several advantages compared to conventional polymer surface modification techniques. Traditional methods, such as plasma or UV treatments, typically provide limited control at the nanoscale and exhibit poor compatibility with chemically inert polymers like PEEK or UHMWPE. By contrast, the BCP method demonstrated in this work enables nanostructuring under relatively mild and straightforward processing conditions, including ambient temperatures, atmospheric pressure, and simple solvent thermal vapor annealing, thus enhancing its scalability and practical applicability for large-area functionalization. Furthermore, the versatility of the BCP self-assembly approach allows deposition of a wide range of metal oxide nanopatterns (e.g., TiO_2_, Al_2_O_3_, MgO, CaO, Ag_2_O), significantly expanding achievable surface chemistries beyond those typically accessible by plasma or UV methods. In comparison to wet chemical techniques such as sol–gel or electrospraying, which often yield coatings with variable thickness, inconsistent nanopatterns, or particle aggregation, the BCP self-assembly method potentially provides more consistent nanopattern formation.

While recognizing some challenges remain, particularly regarding optimization of nanopattern consistency across different metal ions, the BCP self-assembly approach provides a promising alternative to existing surface modification techniques, offering an attractive balance between simplicity, versatility, and achievable nanoscale control.

Additionally, given the chemical composition and properties of the substrates evaluated, similar surface functionalization performance may potentially be obtained when applying this methodology to other polymers with distinct compositions, such as polypropylene (PP), polyaryletherketones (PAEKs), and polyamides like Nylon or Kevlar.

Consequently, this work serves as a proof of concept, paving the way to further exploration and improved understanding of polymer-BCP interactions, as well as optimization of the conditions necessary for obtaining effective self-assembled films and metal oxide nanostructures using diverse block copolymers (BCPs) and a broader variety of metallic ions.

## 3 Experimental Section

### 3.1 Materials

All materials and reagents were used as received without further purification. Semicrystalline, nonchemically treated polyetheretherketone (PEEK) (supplier code: EK303010), quasi-isotropic, nonchemically treated carbon fiber reinforced polyetheretherketone composite (CFPEEK) (supplier code: ET301400 EK40-SH-000110), and ultrahigh molecular weight polyethylene (UHMWPE) (supplier code: ET301400) plates were obtained from Goodfellow Ltd. (U.K.). PEEK, CFPEEK, and UHMWPE were in the form of ≈0.5 mm-thick plates, with the PEEK plate being polished on one side. The plates from all the materials were cut into 10 mm × 10 mm squares which were used as substrates for all experiments. Unless otherwise specified, all the PEEK substrates were treated on their polished side.

The poly(styrene-*b*-ethylene oxide) (PS-*b*-PEO) with molecular weight *M*_w_ = 44,500 g mol^–1^ (*Mn*_*PS*_ = 42,000 g mol^–1^*f%*_*PS*_ = 78.5, *Mn*_*PEO*_ = 11,500 g mol^–1^*f%*_*PEO*_ = 21.5) and polydispersity index PI (*M*_w_/*M*_n_) = 1.06 (supplier code: P4390-SEO) was purchased from Polymer Source Inc. Toluene (TOL)(99.8%, anhydrous), 2-propanol (IPA) (99.5%, anhydrous), Titanium isopropoxide (TTIP) (CAS: 546-68-9), Silver nitrate (AgNO_3_) (CAS: 7761-88-8), Aluminum Chloride (AlCl_3_) (CAS: 7446-70-0), Magnesium nitrate hexahydrate (Mg(NO_3_)_2_·6H_2_O) (CAS: 13446-18-9) and Calcium nitrate tetrahydrate (Ca(NO_3_)_2_·4H_2_O) (CAS: 13477-34-4) were obtained from Sigma-Aldrich U.K. All the listed chemicals were used without further purification.

### 3.2 Methods

#### 3.2.1 Self-Assembled PS-*b*-PEO Thin Film Preparation

The polymer substrates (PEEK, CFPEEK, or UHMWPE) were cleaned by immersion in IPA for 30 min in an ultrasound bath followed by drying in a N_2_ stream. The PS-*b*-PEO was dissolved in TOL to yield a concentration of 3.0 wt %. The substrates were then dip-coated in the PS-*b*-PEO solution for 1 min and removed at a speed of 1 mm/s before drying in air. To obtain a self-assembled PS-*b*-PEO film, a solvent vapor annealing (SVA) process was conducted as follows: the PS-*b*-PEO-coated substrates together with a vial (50 mm × 12 mm × 4 mm) containing 1 mL of TOL were placed in a 150 mL glass jar completely closed. The jar was then placed in a temperature-controlled oven for 1 h at a temperature of 50 ± 2 °C. After this treatment, the samples were removed from the jars and allowed to dry at room temperature.

#### 3.2.2 Metal Ion Infiltration

1 wt % ethanolic solutions of Ca(NO_3_)_2_·4H_2_O, Mg(NO_3_)_2_·6H_2_O, AgNO_3_, and Al(NO_3_)_3_·9H_2_O were used for calcium, magnesium, silver and aluminum infiltration. Substrates coated with the self-assembled PS-*b*-PEO film were dip-coated in the solution of the salt for 5 min, then removed at a speed of 1 cm s^–1^. Subsequently, the samples were rinsed with EtOH and dried in flowing N_2_. For the titanium infiltration, TTIP was used. The PS-*b*-PEO coated substrates were attached facing down to the caps of glass vials (19 mm o̷ × 25 mm height) filled with 0.5 mL of TTIP. The capped vials were then located in a vacuum oven during 20 min at a temperature of 30 ± 2 °C. Afterward, the samples were removed from the vials, then rinsed with IPA and dried with flowing N_2_.

#### 3.2.3 Production of the Metal Oxide Nanopillars

To produce calcium oxide (CaO), magnesium oxide (MgO), aluminum oxide (Al_2_O_3_), silver oxide (Ag_2_O) and titanium oxide (TiO_2_) nanopillar coatings, the metal infiltrated PS-*b*-PEO coated substrates were placed inside a UV ozone (UVO) cleaner system (Novascan PSD Pro-series with a UV lamp with 185 and 254 nm emission wavelength) and irradiated for 3 h at a distance of 11 mm from the UV lamp to remove all the PS-*b*-PEO template and promote the oxidation of the infiltrated metal ions (Figure 1).

## 4 Characterization

### 4.1 Atomic Force Microscopy (AFM)

AFM images were obtained using a Park XE-100 system in noncontact mode (NCM) with an AC160TS cantilever type, which has a force constant of 26 N· *m*^–1^ and a resonance frequency of 300 kHz. All the data processing to obtain Fast Fourier Transform (FFT) images, PSD functions and pore size distribution parameters was conducted using WSxM software,^68^ while the calculation of roughness profiles was obtained using Gwyddion 2.62 software.^69^

### 4.2 Scanning Electron Microscopy (SEM)

Images were recorded on a Zeiss Ultra Plus system with accelerating voltages of 2–10 kV, a working distance from 4 to 10 mm, and a secondary electron detector. All the data processing for the SEM images was carried out using the software ImageJ.^70^

### 4.3 X-ray Photoelectron Spectroscopy (XPS)

XPS analysis was performed under ultrahigh vacuum conditions (<5 × 10^–9^ mbar) with a nonmonochromated source of Al Kα X-rays (1487 eV) and Mg Kα X-rays (1254 eV) operating at 200 and 173 W, respectively (CTX400, PSP Vacuum Technology). The emitted photoelectrons were collected at a takeoff angle of 90° from the sample surface and analyzed in a RESOLVE120 spectrometer (PSP Vacuum Technology). XPS spectra were recorded by setting the analyzer pass energies constant to 100 and 50 eV, for the survey and core scans, respectively. The peak positions of the photoemission lines were corrected to the C(1s) transition, at a binding energy of 285 eV.

## 5 Conclusions

A simple and versatile method has been developed for producing highly ordered metal oxide nanostructures on PEEK, CFPEEK, and UHMWPE polymeric substrates by infiltrating PS-*b*-PEO thin films. We have successfully demonstrated the production of self-organized PS-*b*-PE thin films on high-performance plastics. These thin films have proven to be effective for liquid-phase infiltration syntheses of Al_2_O_3_, MgO, CaO, and Ag_2_O, as well as vapor-phase infiltration of TiO_2_. The resulting metal oxide nanopillars on the evaluated polymeric substrates exhibit diameters on the order of 20 nm, covering a significant portion of the imaged areas. The approach presented in this study showcases the great potential for surface nanostructuring of both high-performance and commercial polymers using metal oxide coatings. The presented methodology could be used for the development of materials applicable in various fields, including biointegration improvement, electronics, photocatalysis, and other areas.

## Supporting Information Available

Additional experimental details and figures, including size distribution histograms, roughness profiles, AFM images of metal oxide coatings, and tables containing Hansen’s solubility parameters, XPS fitting details, and contact angle/surface energy data, together with an explanation of the model used for the calculation of the surface energy (PDF)
