# Supplementary material for: Functionalizing Nonfunctional Surfaces: Creation of Metal Oxide Nanopatterns on High-Performance Polymers via Self-Assembly of PS-b-PEO
Source: ACS Appl Mater Interfaces. 2025 Apr 9;17(16):24654–64. doi: 10.1021/acsami.5c04225 (PMC12022950; doi:10.1021/acsami.5c04225)
Supplement: Supplementary file 1 — am5c04225_si_001.pdf [file am5c04225_si_001.pdf]

Supporting Information

Functionalizing Non-Functional Surfaces: Creation of Metal Oxide Nanopatterns on High-Performance Polymers via Self-Assembly of PS-b-PEO

Jhonattan Frank Baez Vasquez[†], Aislan Esmeraldo Paiva[†], Sajan Singh[†], Sherly Acosta-Beltrán[†], Alberto Alvarez Fernandez[†], and Michael A. Morris[†*]

AMBER Research Centre/School of Chemistry, Trinity College Dublin, Dublin D02W085, Ireland

E-mail: baezj@tcd.ie;mmorris2@tcd.ie

Supporting Information Available

Additional experimental details and figures, including size distribution histograms, roughness profiles, AFM images of metal oxide coatings, and tables containing Hansen's solubility parameters, XPS fitting details, and contact angle/surface energy data, together with an explanation of the model used for the calculation of the surface energy.

Table S1: Hansen solubility parameters used for selecting the solvent used it the solvent vapor annealing step*

**Species**	δ_d	δ_p	δ_h
Styrene	18.6	1.0	4.1
Ethylene Oxide	15.6	10.0	11.0
Toluene	18.0	1.4	2.0

*Parameters obtained from Hansen Solubility Parameters: A User's Handbook, 2007

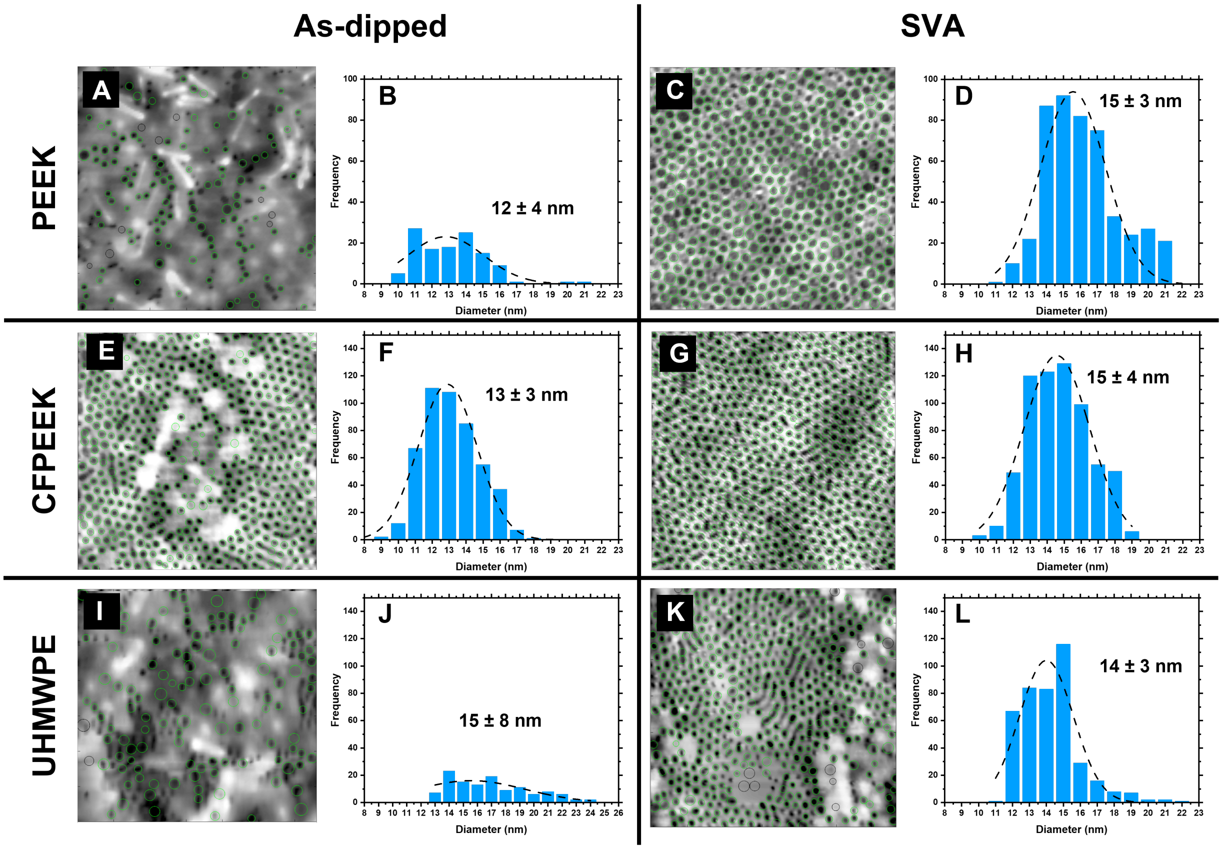

Figure S1: PEO vertical cylinder size distribution for PS-*b*-PEO-coated substrates before and after solvent vapor annealing (SVA): PEEK (A–D), CFPEEK (E–H), and UHMWPE (I–L). Panels A, E, and I show AFM images with particle masks of the as-dipped films; panels B, F, and J show the corresponding size distribution histograms. Panels C, G, and K show AFM images after SVA, with panels D, H, and L presenting the corresponding histograms. Left-side panels show the masked AFM images used to extract size data, while right-side panels show the resulting diameter distributions.

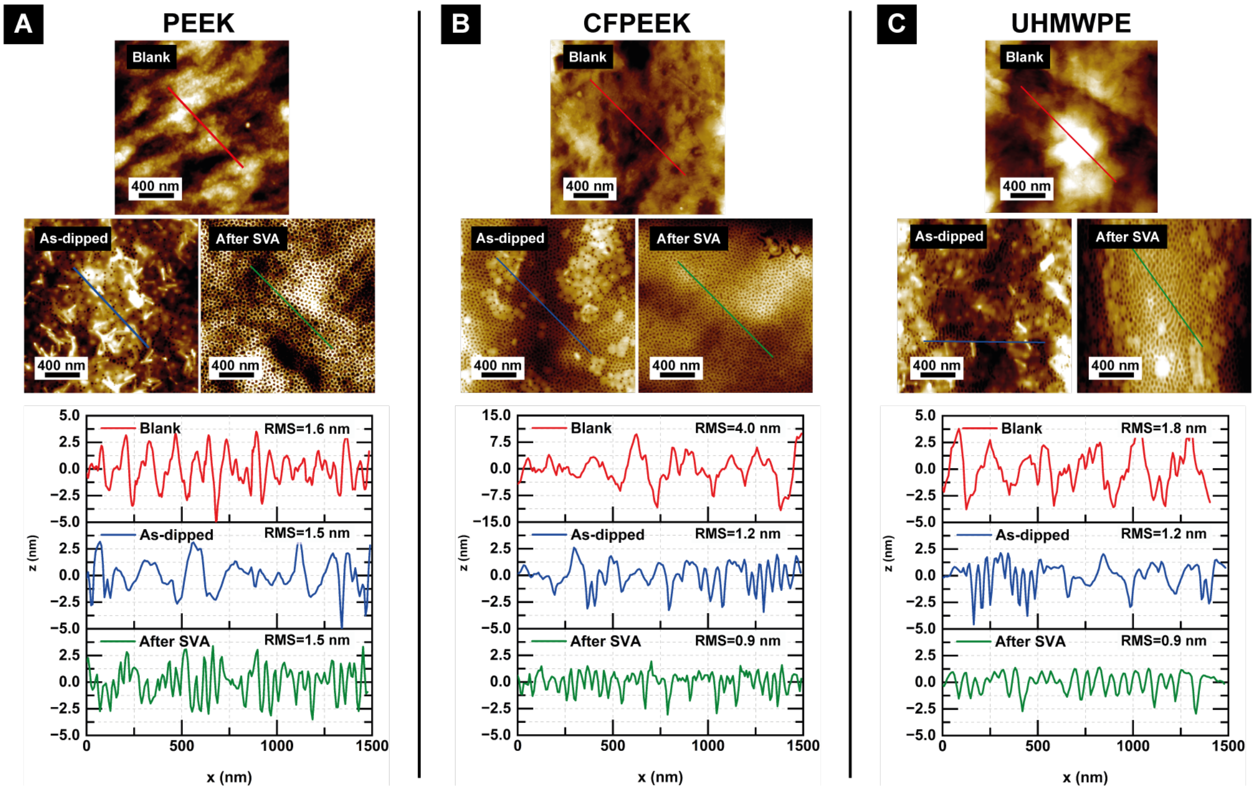

Figure S2: Roughness profiles for PEEK (*a.*), CFPEEK (*b.*) and UHMWPE (*c.*) in the different stages of the production of self-assembled PS-*b*-PEO thin film coatings on each substrate. At the top are the AFM images used for determining the roughness profiles shown at the bottom of each panel. Each colored line in the AFM images matches the color of the line plot shown in the Roughness profiles.

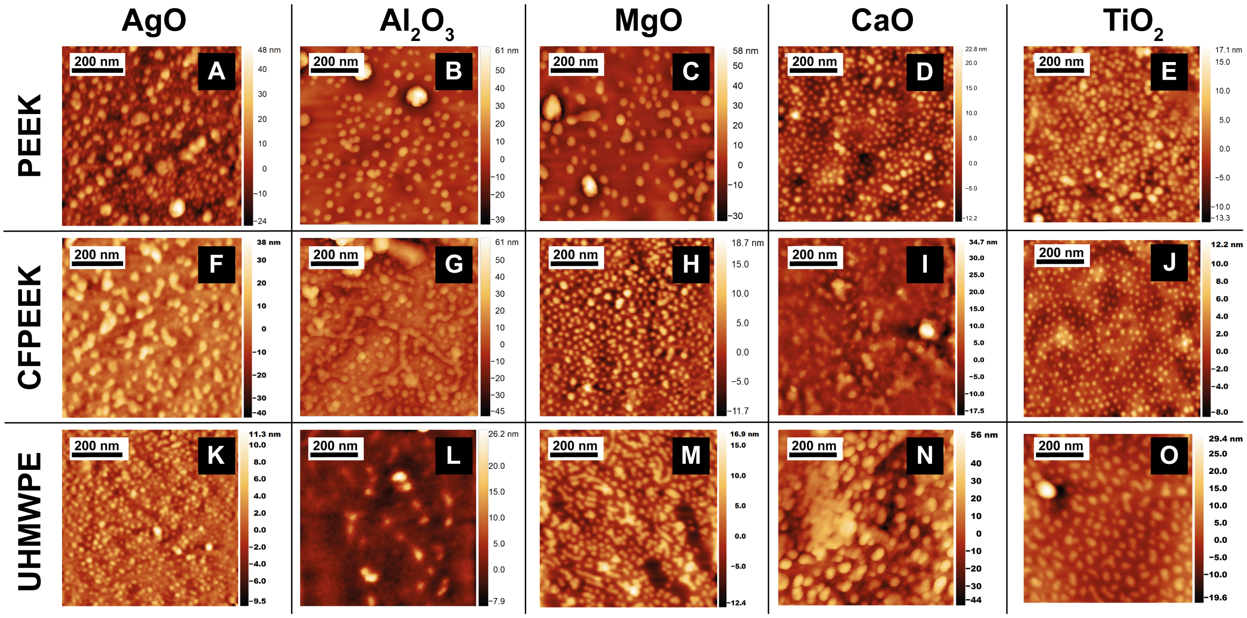

Figure S3: AFM images of metal oxide nanopillar coatings on PS-*b*-PEO templated substrates: PEEK (A–E), CFPEEK (F–J), and UHMWPE (K–O). Columns represent different metal oxides deposited after infiltration and UVO treatment: Ag_2O, Al_2O_3, MgO, CaO, and TiO_2 (left to right). Each image illustrates the resulting nanoscale surface morphology, highlighting differences in pattern formation across substrate types and metal oxide compositions.

Table S2: XPS fitting details for the spectra presented in Figure 8 in the main manuscript

Substrate	Name	Position (eV)	FWHM	L.Sh.
**UHMWPE**	**Ag(I)** $2p_{5/2}$	**368.25**	1.99	GL(40)
	Ag(I) $2p_{3/2}$	374.25	1.99	GL(40)
	Ag(III) $2p_{5/2}$	**369.22**	2.44	GL(30)
	Ag(III) $2p_{3/2}$	375.22	2.44	GL(30)
**PEEK**	**Ag(I)** $2p_{5/2}$	**368.4**	2.44	GL(40)
	Ag(I) $2p_{3/2}$	374.4	2.44	GL(40)
	Ag(III) $2p_{5/2}$	**367.59**	1.54	GL(30)
	Ag(III) $2p_{3/2}$	373.59	1.54	GL(30)
**CFPEEK**	**Ag(I)** $2p_{5/2}$	**369.45**	2.37	GL(50)
	Ag(I) $2p_{3/2}$	375.45	2.37	GL(50)
	Ag(III) $2p_{5/2}$	**368.53**	1.87	GL(30)
	Ag(III) $2p_{3/2}$	374.53	1.87	GL(30)
**UHMWPE**	**Al(III) 2p**	**75.41**	2.08	GL(30)
**PEEK**	**Al(III) 2p**	**75.07**	2.29	GL(30)
**CFPEEK**	**Al(III) 2p**	**75.34**	2.19	GL(30)
**UHMWPE**	**Ca(II)** $2p_{3/2}$	**346.66**	2.19	GL(30)
	Ca(II) $2p_{1/2}$	350.21	2.19	GL(30)
**PEEK**	**Ca(II)** $2p_{3/2}$	**346.97**	2.21	GL(30)
	Ca(II) $2p_{1/2}$	350.52	2.21	GL(30)
**CFPEEK**	**Ca(II)** $2p_{3/2}$	**347.29**	2.43	GL(30)
	Ca(II) $2p_{1/2}$	350.84	2.43	GL(30)
**UHMWPE**	**Mg(II) 2p**	**50.74**	2.55	GL(30)
**PEEK**	**Mg(II) 2p**	**50.3**	2.85	GL(30)
**CFPEEK**	**Mg(II) 2p**	**50.851**	2.53	GL(30)
**UHMWPE**	**Ti(IV)** $2p_{3/2}$	**458.9**	1.98	GL(30)
	Ti(IV) $2p_{1/2}$	464.6	2.63	GL(30)
	Shake-up	461.37	2.13	GL(30)
**PEEK**	**Ti(IV)** $2p_{3/2}$	**459.11**	1.95	GL(30)
	Ti(IV) $2p_{1/2}$	464.81	2.67	GL(30)
	Shake-up	461.2	1.81	GL(30)
**CFPEEK**	**Ti(IV)** $2p_{3/2}$	**458.9**	2.32	GL(30)
	Ti(IV) $2p_{1/2}$	464.6	2.45	GL(30)
	Shake-up	462	2.5	GL(30)

Table S3: Contact angle and surface energy parameters for different substrates. WCA: water contact angle; Cl_2H_2: contact angle of the second liquid (diiodomethane); γ_s^d: dispersive component; γ_s^p: polar component; γ_s: total surface energy.

**Substrate**	**WCA (°)**		**Cl_2H_2 (°)**		γ_s^d (mJ/m^2)		γ_s^p (mJ/m^2)		γ_s (mJ/m^2)	
	**Val.**	±	**Val.**	±	**Val.**	±	**Val.**	±	**Val.**	±
PEEK	92.58	1.24	75.30	1.29	16.87	0.99	4.72	0.82	21.60	1.29
CFPEEK	101.32	0.75	53.14	4.50	32.06	3.28	0.15	0.20	32.21	3.28
UHMWPE	88.36	1.47	50.09	3.85	29.56	2.86	2.81	1.02	32.37	3.04

Surface energy γ_s was determined using the Owens-Wendt-Rabel-Kaelble (OWRK) model. This method splits γ_s into dispersive (γ_s^{d}) and polar (γ_s^{p}) components by measuring contact angles θ with two probe liquids (e.g., water and diiodomethane), each having known dispersive and polar contributions. The key equation is given by

$$\gamma_l\big(1+\cos\theta\big) = 2\Big(\sqrt{\gamma_s^{\mathrm{d}}\,\gamma_l^{\mathrm{d}}} \;+\; \sqrt{\gamma_s^{\mathrm{p}}\,\gamma_l^{\mathrm{p}}}\Big),$$

where γ_l is the liquid surface tension (also broken down into dispersive γ_l^{d} and polar γ_l^{p} parts). Solving the resulting system by linearization, arrangement into a 2×2 system of equations, and matrix-based operations yields γ_s^{d} and γ_s^{p}. The total surface energy is then computed as

$$\gamma_s = \gamma_s^{\mathrm{d}} + \gamma_s^{\mathrm{p}}.$$

All measurements shown on Table S3 were conducted at room temperature, and each contact angle value represents the average of at least three advancing contact angle measurements.
